# Time trends and regional variations in prices of anticancer medicines in China

**DOI:** 10.3389/fphar.2024.1397784

**Published:** 2024-05-15

**Authors:** Jinwei Zhang, Shuchen Hu, Xingyu Liu, Xiaoyong Liu, Jieqiong Zhang, Caijun Yang, Yu Fang

**Affiliations:** ^1^ Department of Pharmacy Administration and Clinical Pharmacy, School of Pharmacy, Xi’an Jiaotong University, Xi’an, China; ^2^ Center for Drug Safety and Policy Research, Xi’an Jiaotong University, Xi’an, China

**Keywords:** cancer, medicine price, time trends, regional variation, China

## Abstract

**Introduction:**

High prices, as a main factor, contributed to the lack of adequate access to essential anticancer medicines, especially for patients in developing countries. The Chinese Government has introduced a series of policies to control the prices of medicines during the last decade, but the effect on anticancer medicine is not yet clear.

**Methods:**

To evaluate the time trends and regional variation in the price of essential anticancer medicines in China, we used the procurement data of anticancer medicines from 2015 to 2022. We selected 29 anticancer medicines from the 2018 Chinese National Essential Medicines List. To measure the cost of a medicine, we used defined daily dose cost -the cost per defined daily doses. At national level, we focused on the price changes over time and compared the price between medicine categories. At provincial level, we assessed price variation among provinces over time.

**Results:**

For prices at the national level, all 6 targeted medicines exhibited a continuous decrease trend in price. Out of 23 non-targeted medicines, 4 (17·39%) experienced continuous increases in prices, and 9 (39·13%) showed price decreases from 2015 to 2019 and then an upward trend during 2019–2022; Of the remaining non-targeted medicines, 7 (30·43%) had continuous price decreases and 3 (13.04%) had price increases followed by decreases. For prices at the provincial level, provincial price variation became smaller for almost all targeted medicines, except rituximab; for 11 out of 23 non-targeted medicines, provincial price variations became larger. During the study period, the proportion of price-increased medicines in each province was geographically correlated, and no significant relationship between prices and GDP *per capita* was observed for both targeted and non-targeted anticancer medicines.

**Conclusion:**

The prices and regional disparity of most targeted anticancer medicines were decreasing, while for nearly half of the non-targeted anticancer medicines, the prices were increasing and the regional disparity became wider, which may lead to compromised access to these essential anticancer medicines and raise inequity health outcome among regions.

## Introduction

Cancer has become a prominent cause of death globally. In 2020, it accounted for nearly 10 million deaths worldwide (World Health Organization, 2023). To reduce the growing burden of cancer, ensuring universal access to essential cancer treatments is one of the key approaches. The World Health Organization’s Model list of essential medicines included dozens of anticancer medicines, which provided a reference to countries to develop their own national essential anticancer medicines. However, the affordability of the essential anticancer medicines, especially that of novel targeted therapies, challenged many low- and middle-income countries (World Health Organization, 2018; Bygrave et al., 2021).

As the world’s largest developing country, China has a substantial cancer burden. In 2020, there were 4·57 million new cancer cases and 3 million cancer deaths in China, accounting for 23.7% of new cancer cases and 30% of cancer deaths in the whole world, respectively (Qiu et al., 2021). According to a study based on 1,608 Chinese cancer survivors, about half of survivors borrowed money or went into debt because of cancer and approximately 10% of these survivors reported forgoing medical care because of cost; The high cost of cancer medicines is a major barrier (Su et al., 2020; Qiu et al., 2021).

To respond to this issue, Chinese government implemented a series of innovative policies since 2009 (Nation Health Commission of the People’s Republic of China, 2023a). The National Essential Medicine List was formulated in 2009, and updated in 2012 and 2018 (Central People’s Government of the People’s Republic of China, 2009a; Central People’s Government of the People’s Republic of China, 2012; National Health Commission of the People’s Republic of China, 2018). The three lists contained 307 (zero anticancer medicines), 520 (26 anticancer medicines using in chemotherapy and endocrine therapy) and 685 medicines (35 anticancer medicines, 29 using in chemotherapy and endocrine therapy and 6 targeted medicines), respectively. In 2009, a province-based competitive-bidding system for medicines was established to obtain low prices; manufacturers bid in different provinces and medicines were procured for the whole province at agreed bid prices (National Health Commission of the People’s Republic of China, 2023b). To reduce the medicine expenditure in health institutions, the government mandated the Zero-Markup Drug Policy (eliminating the 15% mark-up allowance on prescribed drugs) for primary health institutions in 2009, for county-level hospitals in 2012, and for city-level hospitals in 2015 (Central People’s Government of the People’s Republic of China, 2009b; Central People’s Government of the People’s Republic of China, 2023b; GOV, 2023a). To promote accessibility of innovative medicines, the government initiated the national medication price negotiation (NMPN) in 2016 and followed annually thereafter. All the successfully negotiated drugs would be included in the national reimbursement drug list (NRDL). In terms of anticancer medicines, the NMPN focused on expensive targeted medicines. Among anticancer medicines in the NMPN, more than three-quarters were targeted medicines during 2017–2020 (National Healthcare Security Administration, 2023a; GOV, 2023b; National Healthcare Security Administration, 2023b; NHSA, 2023).

The Lancet Commission on Essential Medicines called for continuous and global monitoring of access to essential medicines (including anticancer medicines) (Tanimoto et al., 2017; Simão et al., 2018). Several studies have documented increasing trends in the use and costs of anticancer medicines in developed countries (Goldstein et al., 2017; Salmasi et al., 2017; Rachamin et al., 2021; Vokinger et al., 2022). Three studies found that the use of anticancer medicines increased over time in China (Hwang et al., 2019; Pan et al., 2022; Zhu et al., 2022). However, the evidence on prices of essential anticancer medicines in China is limited.

Considering the Chinese central government’s efforts to control the prices of anticancer medicines and provincial implementation of medicine procurement, we aimed to assess the time trends and regional variation in price of essential anticancer medicines in China using the procurement data of anticancer medicines from 2015 to 2022.

## Materials and methods

### Data sources and extraction

We considered the 35 anticancer medicines in the 2018 Chinese National Essential Medicines List. Two adjuvant medicines (mesna and ondansetron) with multiple anatomical therapeutic chemical (ATC) codes were excluded.

To measure the cost of a medicine, we used defined daily dose cost (DDDC)-the cost per defined daily doses (DDDs). The defined daily dose of a medicine is the assumed average maintenance dose per day for the medicine used for its main indication in adults (WHO Collaborating Centre for Drug Statistics Methology, 2023). Consistent with our previous research, we used defined daily dose values provided by the German Federal Institute for Drugs and Medical Devices (Pan et al., 2022; Bonn, 2023). Where we could not obtain a defined daily dose for a medicine, we used the daily dose for the main treatment in the medicine’s product information as a reference. We excluded tretinoin and methotrexate because they both have multiple ATC codes and multiple DDDs.

To calculate the DDDC for each medicine, we collected annual procurement data for the studied anticancer medicines from 2015 to 2022 from 31 provincial National Centralized Drug Procurement service centers in China. The data included the generic name, chemical substance name, dosage form, strength, purchase amount and quantity of the medicine. Two medicines were excluded because of implausible procurement data. Finally, 29 anticancer medicines were selected, including 23 non-targeted and 6 targeted medicines (Table 1). For all the 29 medicines, we divided the total quantity of each medicine purchased by its defined daily dose to estimate the number of defined daily doses procured per year. The DDDC is calculated by dividing the total expenditure of each medicine by its number of defined daily doses procured per year. All the prices were adjusted to 2022 level using the Consumer Price Index (CPI) (1 US dollar = 6.726 RMB in 2022) (National Bureau of Statistics of the People’s Republic of China, 2023).

**TABLE 1:** National relative price changes, categories and defined daily dose of anticancer medicines.

	Categories	Medicine	Defined daily dose (mg)	Relative price change (2015–2019) (%)	*p*-Value	Relative price change (2019–2022) (%)	*p*-Value	Types of price changes (2015–2019–2022)
Non-targeted anticancer medicines	Alkylating agent	Busulfan	224	−9 · 95	0.052	−5 · 85	0.784	Continuous decrease
		Cyclophosphamide	50 (oral)	260·17		122·55		Continuous increase
			250 (parenteral)					
		Ifosfamide	700	−51·36		17·08		Decrease followed by increase
	Anticancer antibiotics	Daunorubicin	7	−12·45		−29·40		Continuous decrease
		Doxorubicin	5	−23·94		34·26		Decrease followed by increase
		Etoposide	50 (oral)	−65·99		228·95		Decrease followed by increase
			25 (parenteral)					
	Anticancer botanical ingredients	Homoharringtonine	1	947·96		−2 · 72		Increase followed by decrease
		Taxol	15	−49·04		9·79		Decrease followed by increase
		Vincristine	0.36	406·17		270·45		Continuous increase
	Anticancer hormones	Letrozole	2.5	−24·34		−72·07		Continuous decrease
		Tamoxifen	20	40·52		57·95		Continuous increase
	Antimetabolite	Cytarabine	50	−52·85		72·25		Decrease followed by increase
		Fluorouracil	150 (oral)	200·51		−23·72		Increase followed by decrease
			100 (parenteral)					
		Gemcitabine	200	−38·94		−65·18		Continuous decrease
		Hydroxyurea	1750	15·87		51·64		Continuous increase
		Mercaptopurine	175	168·05		−5 · 06		Increase followed by decrease
	Other anticancer medicines	Arsenous acid	6.19	−3 · 87		−5 · 12		Continuous decrease
		Asparaginase	5000	−11·53		−11·60		Continuous decrease
		Calcium folinate	60	−59·12		45·65		Decrease followed by increase
		Capecitabine	3000	−60·24		−70·42		Continuous decrease
		Carboplatin	25	−35·27		83·81		Decrease followed by increase
		Cisplatin	6.75	−36·55		5·42		Decrease followed by increase
		Oxaliplatin	11	−71·83		121·16		Decrease followed by increase
Targeted anticancer medicines		Alimta	43	−49·48	0.028*	−55·90	0.028*	Continuous decrease
		Gefitinib	250	−86·21		−64·27		Continuous decrease
		Icotinib	125	−54·16		−41·57		Continuous decrease
		Imatinib	400	−76·39		−61·52		Continuous decrease
		Rituximab	32	−47·64		−28·99		Continuous decrease
		Trastuzumab	20	−71·30		−29·86		Continuous decrease

Note: *p < 0.05.

### Analysis

For each province, if it has less than 15 medicines with price data in 1 year, then all the price information of this province in that year would be excluded in the following analysis (Supplementary Table S1). Based on the provincial information, we calculated the provincial-level and national-level price for each medicine.

At national-level, we focused on the price changes over time and differences between medicine categories. The National Healthcare Security Administration of China (NHS) was established in March 2018 and was responsible for formulating bidding and procurement policies for medicines. Since December 2018, NHS has organized several national drug price negotiations (involving more anticancer medicines than previous national negotiations), which had a significant impact on anticancer medicine prices (Xu et al., 2019; National Healthcare Security Administration, 2023a; National Healthcare Security Administration, 2023b; NHSA, 2023). Considering that, we calculated the relative price changes for three periods (2015–2019, 2019–2022 and 2015–2022) for each medicine; and based on this information, we classified the trends of medicine prices into four types: continuous decrease, decrease followed by increase, continuous increase and increase followed by decrease. Wilcoxon test was used to explore the significance of price changes for targeted and non-targeted drugs over 2015–2019 and 2019–2022. We also categorized the 29 medicines as targeted therapies medicines (referred as targeted medicines) and traditional chemotherapy and endocrine therapy medicines (referred as non-t argeted medicines for comparison purposes) to explore possible differences in price trends between them.

At provincial-level, we assessed price variation among provinces over time. For a medicine, we defined the provincial price variation as the gaps between its highest and lowest provincial price in a specific year. By comparing the provincial price variation in 2015 and 2022, we could describe whether the provincial price variation for a medicine was increasing or decreasing over time. In a province, we compared the price change between the first and last year for each medicine during 2015–2022. The proportion of the price-increased medicines in each province was calculated. To picture the provincial difference, all the provinces were divided into four tiers based on the four equal parts of their proportions. Moreover, we performed two sensitivity analyses to ensure the robustness of this result. First, we only considered medicines whose price data were available in all provinces (Tibet was excluded because it used fewer medicines than other provinces). Second, in a province, we only considered medicines whose price data were available in 2015 and 2022 (the overlap of procured anticancer medicines in 2015 and 2022 were low for both Qinghai and Ningxia provinces, therefore we excluded these two provinces).

Scatterplots were utilized to explore the relationship between provincial median price of anticancer medicines and *per capita* Gross Domestic Product (GDP) in 2022.

## Results

### Price trends of anticancer medicines at national level

**FIGURE 1:**
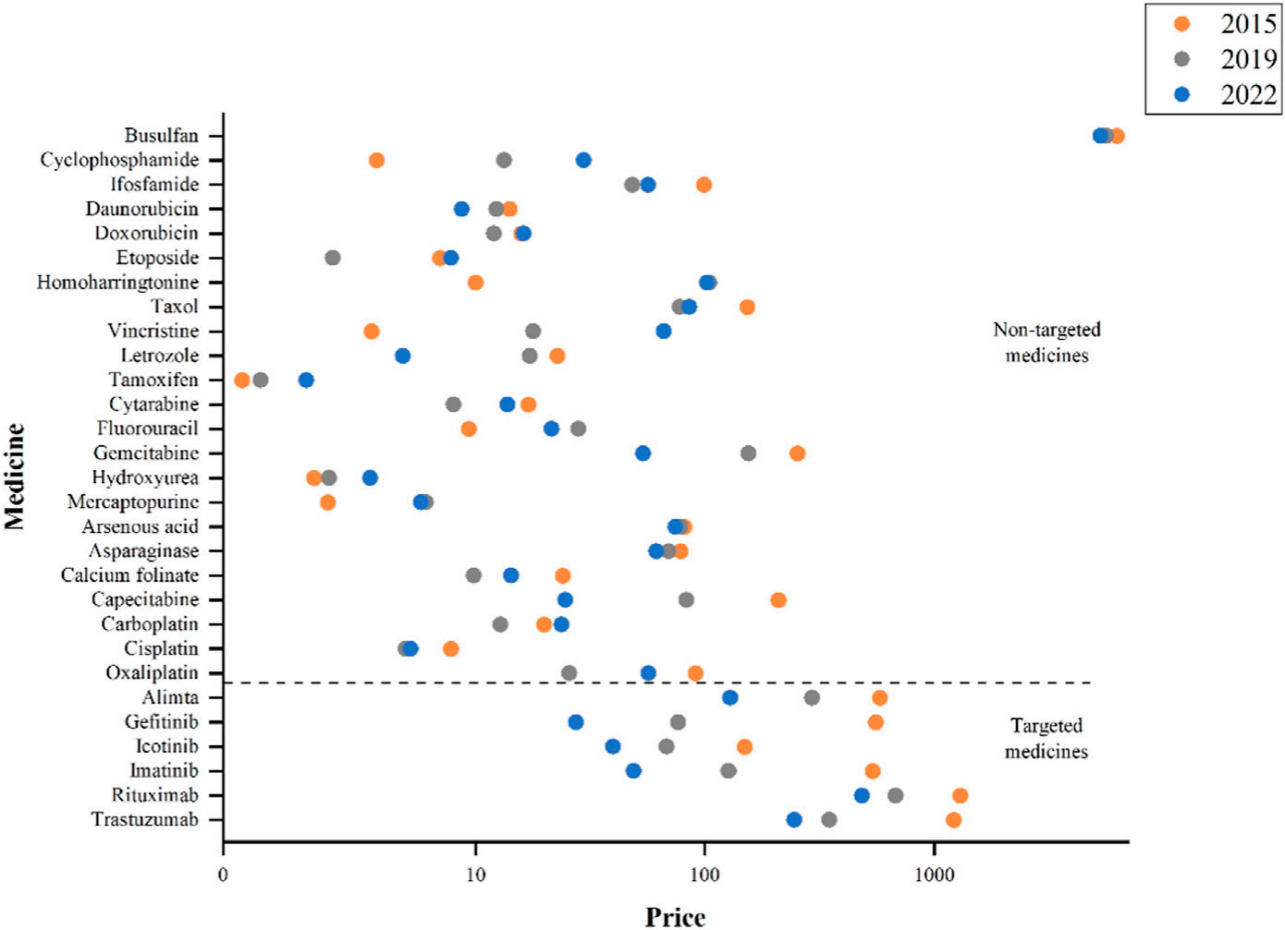
National prices of anticancer medicines in 2015, 2019, and 2022.

The national-level prices of 29 medicines in 8 years (2015–2022) were in Supplementary Table S2. The price trends of targeted and non-targeted medicines were quite different via comparing the prices in 2015, 2019, and 2022 (Figure 1). All the targeted medicines showed substantial price declines, but were still at a higher price compared to non-targeted medicines. Among the 6 targeted medicines, rituximab and trastuzumab had comparatively higher prices; while among the 23 non-targeted medicines, busulfan has remained at the highest price in 2015, 2019, and 2022 despite with a downward trend.

During 2015 to 2019, 7 out of 23 non-targeted medicines had an increase in price. In terms of the relative price changes, the top three were homoharringtonine (947·96%), vincristine (406·17%) and cyclophosphamide (260·17%). While, during 2019–2022, the price of homoharringtonine dropped slightly (−2.72%), and the prices of vincristine (270·45%, ranked first in 2019–2022) and cyclophosphamide’s (122·55%, ranked third in 2019–2022) continued to rise. Totally, 13 non-targeted medicines experienced increase in price. The top three non-targeted medicines with largest price reduction from 2015 to 2019 were oxaliplatin (−71·83%), etoposide (−65·99%) and capecitabine (−60·24%). During 2019 to 2022, capecitabine (−70·42%) continued to decrease in price, while etoposide (228·95%, ranked second in 2019–2022) and oxaliplatin (121·16%, ranked fourth in 2019–2022) experienced significant price increases (Table 1).

Overall, all the six targeted medicines exhibited a continuous decrease trend in price changes. Out of 23 non-targeted medicines, 4 (17·39%) experienced continuous increase in prices (Vincristine, Cyclophosphamide, Hydroxyurea and Tamoxifen), and 9 (39·13%) showed price decrease during 2015–2019 and then an upward trend during 2019–2022. For targeted medicines, the prices dropped significantly from 2015 to 2019 (*p* = 0.028) and 2019 to 2022 (*p* = 0.028), but the results for non-targeted drugs were not significant (Table 1).

**FIGURE 2:**
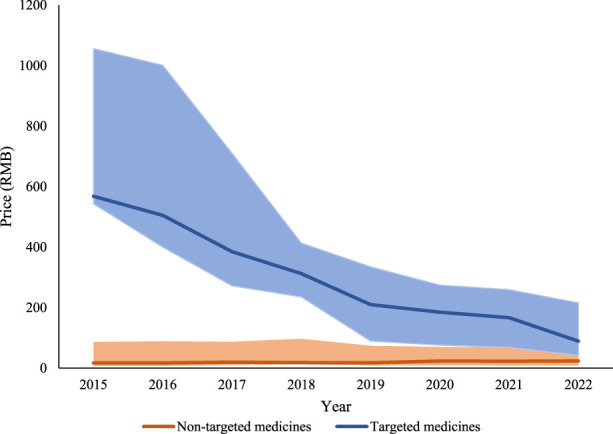
Price for targeted and non-targeted medicines from 2015 to 2022. Note: The solid line represents the median prices. The lower end and the upper end of the shaded area represents the first and the third quartile prices, respectively.

The time trend in prices of targeted medicines and non-targeted medicines were displayed in Figure 2. The median price of six targeted medicines decreased significantly (*p* = 0·28), from 494.46 in 2015 to 77·52 in 2022; while, the median price of 23 non-targeted medicines increased slightly (*p* = 0·14), from 14·31 in 2015 to 21·03 in 2022.

### Provincial variation in prices of anticancer medicines

The provincial price variations for all the medicines in 2015 and 2022 were in Table 2. In 2015, the three medicines with the largest price variation were imatinib (targeted medicine, 817.51), taxol (non-targeted medicine, 500·09) and gemcitabine (non-targeted medicine, 460·02). In 2022, the provincial price variation of imatinib still ranked the first (443·37), doxorubicin ranked the second (non-targeted medicine, 337·24) and tituximab ranked the third (targeted medicine, 313·28). Overtime, provincial price variation became smaller for almost all targeted medicines, except rituximab (+48·58); While, for 11 out of 23 non-targeted medicines, provincial price variations became larger (Supplementary Figure S1).

**TABLE 2:** Provincial price variations of anticancer medicines.

Non-targeted anticancer medicines	Price variation (2015)	Price variation (2022)	Changes in price variation	Non-targeted anticancer medicines	Price variation (2015)	Price variation (2022)	Changes in price variation	Targeted anticancer medicines	Price variation (2015)	Price variation (2022)	Changes in price variation
Busulfan	0	115·02	115·02	Fluorouracil	428·14	305·78	−122·36	Alimta	336·39	277·24	−59·15
Cyclophosphamide	17·17	8·68	−8 · 49	Gemcitabine	460·02	220·01	−240·01	Gefitinib	155·13	144·54	−10·59
Ifosfamide	258·58	99·03	−159·55	Hydroxyurea	2·77	3·64	0·87	Icotinib	32·94	24·25	−8 · 69
Daunorubicin	49·08	5·97	−43·11	Mercaptopurine	0·32	1·23	0·91	Imatinib	817·51	443·37	−374·14
Doxorubicin	42·4	337·24	294·84	Arsenous acid	22·11	3·16	−18·95	Rituximab	264·7	313·28	48·58
Etoposide	31·66	144·37	112·71	Asparaginase	29·4	20·21	−9 · 19	Trastuzumab	191·59	15·53	−176·06
Homoharringtonine	42·38	68·66	26·28	Calcium folinate	65·68	109·4	43·72				
Taxol	500·09	83·15	−416·94	Capecitabine	212·83	115·2	−97·63				
Vincristine	21·65	50·39	28·74	Carboplatin	24·23	28·67	4·44				
Letrozole	22·7	33·65	10·95	Cisplatin	34·16	2·92	−31·24				
Tamoxifen	0·96	4·09	3·13	Oxaliplatin	200·2	115·31	−84·89				
Cytarabine	16·56	5·63	−10·93								

**FIGURE 3:**
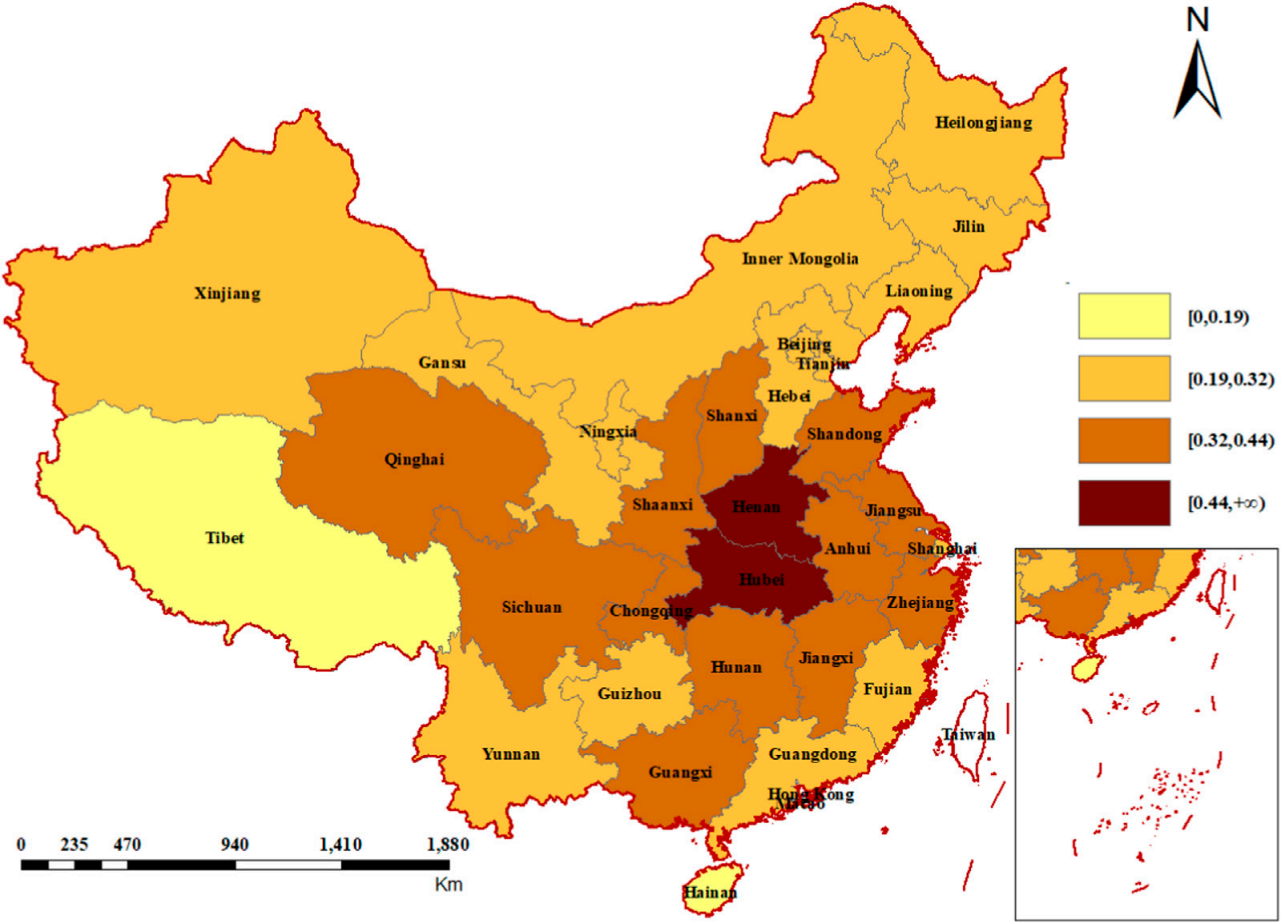
Proportion of the price-increased medicines in provinces, 2015–2022.

The proportion of the price-increased medicines in each province was displayed in Figure 3. Henan and Hubei, two central provinces, are the first-tier part with highest percentage of price-increased medicines. Generally, the second-tier provinces were situated around the first-tier provinces, and the third-tier provinces encompassed the second tier. Hainan and Tibet were in the fourth echelon, with the lowest proportion of price-increased medicines. Two sensitivity analyses showed similar results (Supplementary Figures S2, S3).

**FIGURE 4:**
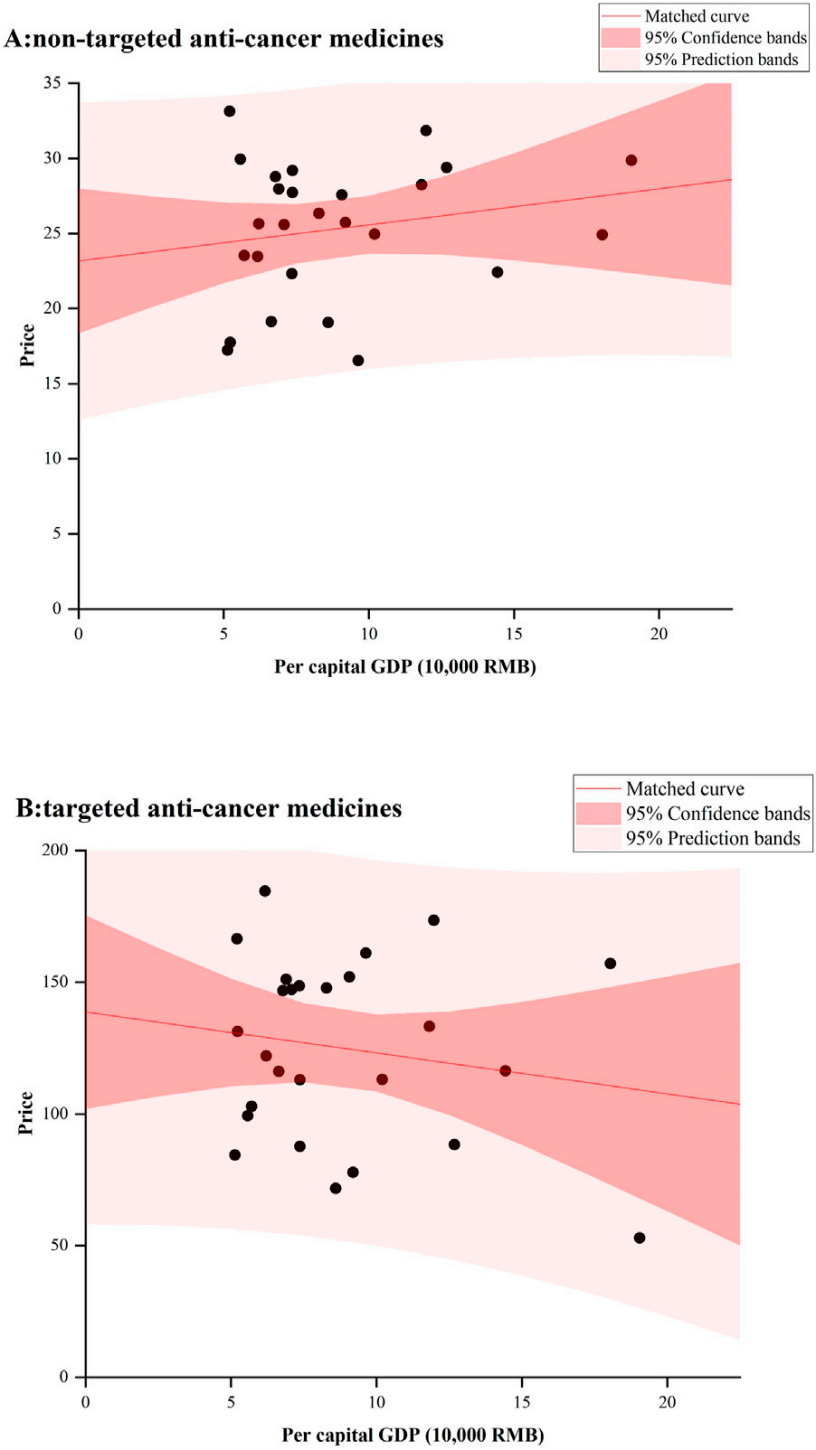
Correlation between medicine prices and GDP *per capita*, by Chinese province, 2022.

Figure 4 showed the relationship between provincial anticancer medicines’ prices and GDP in 2022. No significant results were observed for both targeted and non-targeted medicines. For non-targeted medicines, some underdeveloped provinces had very high median prices, such as Guangxi, Hunan, and Jilin; While for targeted medicines, Beijing with the highest GDP had the lowest median price.

## Discussion

Access to affordable, high-quality essential medicines is crucial for countries aiming to achieve universal health coverage. The Chinese government has made a lot of efforts to promote access to affordable medicines during last decade, especially for medicines treating cancer. To evaluate the effect, our study measured the time trends and regional variation in price of essential anticancer medicines at the national and provincial levels in China during 2015–2022.

Nationally, all 6 targeted medicines decreased greatly in prices, while 4 (17.39%) non-targeted medicines experienced continuous increases in prices and 9 (39.13%) had price decreases followed by increases during 2015–2022. With high prices and poor affordability, targeted medicines have been the focus of Chinese government during last several years. National medicine price negotiation which combined with mandatory reimbursement (NMPN) has been implemented annually since 2016, and many high-priced medicines were included. Moreover, National Centralized Drug Procurement (NCDP) starting in 2019 has been adopted, and more than 300 kinds of medicines were successfully procured via this policy, containing some targeted medicines. As the largest payer of medical expenditures, Chinese National Healthcare Security Administration has strong bargaining power during the negotiation or the centralized procurement; Medicine manufacturers are willing to cut prices to be included on the reimbursement list or gain a promised market share. All the six targeted medicines and five out of 23 non-targeted medicines were included in the centralized procurement catalog or national drug price negotiation list. The left 18 non-targeted medicines are procured through provincial competitive-bidding system in each province. In addition, anticancer medicines were plagued by shortage issue in China, especially those medicines used in traditional chemotherapy (Drug Shortage, 2023). In our study, homoharringtonine and mercaptopurine were reported in shortage in 2017, and etoposide were in 2021 (Health Commission of Shandong Province and China, 2023; Central People’s Government of the People’s Republic of China, 2023a; Shanxi Provincial Health Commission and China, 2021). Correspondingly, the three medicines showed a price increase in 2017, 2018, and 2021, respectively. Although Chinese government has actively implemented a series of strategies since 2017 to alleviate the shortage issue, the price hike pressure caused by the shortages has not been relieved even after the resolution of shortages. More effective policies should be proposed; Otherwise, high prices would be another barrier to access to these medicines.

Regional variation in prices were decreasing for all targeted medicines (except rituximab), and increasing for nearly half of the non-targeted medicines (11/23). Differentiated procurement policies for different medicines mainly contributed to this result. All six targeted medicines and most non-targeted medicines with significant reduction in regional variation (taxol, −416·94 regional variation changes; gemcitabine, −240·01; capecitabine, −97·63) were involved in the national-level procurement policies (including NMPN and NCDP) (Central People’s Government of the People’s Republic of China, 2023c; Central People’s Government of the People’s Republic of China, 2023d; Central People’s Government of the People’s Republic of China, 2023e). National-level policies can create a uniform price for a product across the country as they provided a price reference for medicines produced by different companies, while most non-targeted medicines were just procured through provincial competitive-bidding system. As the price information in provincial systems was not transparent, the price of the same product varied greatly across provinces. Recently, the Chinese National Healthcare Security Administration is establishing a national platform to collect the real-time price and usage information in 31 provinces (National Healthcare Security Administration, 2023). We believe that the issue of price disparity among provinces would be resolved once the price information is shared among the provincial administrations.

The proportion of the price-increased medicines in each province was geographically correlated, in other words, the proportions in neighboring provinces were close. One possible reason was that communication between neighboring provinces is more frequent. Provincial government administrators usually took the performance of neighboring provinces (here is drug price) as target, and patients were more likely to know the price of drugs in neighboring regions. Another reason might be the interprovincial medicine procurement alliances formed for medicines treating specific diseases, which was popular during last several years. For example, a 14-province alliance (consisting of Shaanxi, Inner Mongolia, Ningxia, Gansu, Qinghai, Xinjiang, Hunan, Heilongjiang, Liaoning, Guangxi, Guizhou, Hainan, Shanxi and Jilin) bargained for 47 anticancer medicines in 2018; the Yangtze River Delta Alliance (Shanghai, Zhejiang and Anhui) completed centralized drug procurement in 2022, including imatinib and other anticancer medicines; Gansu and Shaanxi formed alliance for several procurement, covering medicines treating diabetes, hypertension and so on (Interprovincial Union of 14 Provinces, 2023; Yangtze River Delta Alliance, 2023; Gan-Shaan alliance, 2023). Usually, provinces in a league were geographically close to each other. The alliance negotiated with manufactures by pooling the needs of member provinces to obtain a better price. If the negotiation succeeded, the price of the negotiated medicine was consistent across member provinces. The alliance is very flexible, and different procurement alliances can be formed for different medicines. For provinces, it is imperative to sort out their high-priced or high-demand medicines, and form procurement alliances with other provinces (not limited to neighboring provinces) to increase their bargaining power on price negotiation, thereby getting a lower price for these medicines.

There was no significant relationship between prices and GDP *per capita* for both targeted and non-targeted anticancer medicines. Ideally, medicine prices should be lower in provinces with lower GDP *per capita*, comparing that in provinces with higher GDP *per capita*. However, we found that some less developed provinces had higher median prices for anticancer medicines. In addition to weaker bargaining power, some less developed provinces may have procured more expensive brand medicines, which would lead to a high economic burden for patients in these provinces. Similar result was generated in a cross-sectional survey in China, which found that less developed provinces had a higher availability of brand medicines in their private pharmacies (Yang et al., 2020).

Our study has several limitations. First, we only included 29 anticancer medicines on the 2018 NEML, so our findings might not be generalizable outside of the studied medicines. As scheduled, the NEML should be updated every 3 years since 2018. However, the new version of NEML has not yet been published, resulting in some new, widely-used medicines on the market not being included in the study. Furthermore, the number of targeted and non-targeted medicines were not even. Second, we used the procurement data of anticancer medicines in 31 provinces. The prices used in this study were at national level and provincial level and might not reflect the prices paid by patients. Moreover, the basic medical insurance in China is based on the prefecture-level and county-level city as the basic pooling unit, and usually economically developed areas tend to offer higher levels of protection. Therefore, our analysis of price disparity among provinces may underestimate the disparity in the net price. Third, in some years during the study period, there were some provinces where their data were unavailable or incomplete because of multiple reasons (such as update of information system). To ensure the data quality, we only included provinces which has price data for at least 15 medicines in analysis. The actual national price for each medicine might be different. As there were no more than four provinces (31 in total) which were excluded each year, the potential bias would be small.

## Conclusion

This study revealed time trends and regional variation in price of essential medicines to treat cancer from 2015 to 2022 in China. The prices and regional disparity of most targeted anticancer medicines were decreasing, while for nearly half of the non-targeted anticancer medicines, the prices were increasing and the regional disparity became wider, which may lead to compromised access to these essential anticancer medicines and raise inequity health outcome among regions. The Chinese government needs to pay attention to these issues and implement specific policies to improve access to essential non-targeted anticancer medicines and eliminate inequalities across regions.

## Data Availability

The original contributions presented in the study are included in the article/Supplementary Material, further inquiries can be directed to the corresponding authors.
