# Supplementary material for: Time trends and regional variations in prices of anticancer medicines in China
Source: Front Pharmacol. 2024 May 15;15:1397784. doi: 10.3389/fphar.2024.1397784 (PMC11133614; doi:10.3389/fphar.2024.1397784)
Supplement: Supplementary file 1 [file DataSheet1.docx]

**Supplementary Table 1 Numbers of anticancer medicines in year-province data**

| Year | **Anhui** | **Beijing** | **Fujian** | **Gansu** | **Guangdong** | **Guangxi** | **Guizhou** | **Hainan** | **Hebei** | **Henan** | **Heilongjiang** | **Hubei** | **Hunan** | **Jilin** | **Jiangsu** | **Jiangxi** | **Liaoning** | **Inner Mongolia** | **Ningxia** | **Qinghai** | **Shandong** | **Shanxi** | **Shaanxi** | **Shanghai** | **Sichuan** | **Tianjin** | **Tibet** | **Xinjiang** | **Yunnan** | **Zhejiang** | **Chongqing** |
| --- | --- | --- | --- | --- | --- | --- | --- | --- | --- | --- | --- | --- | --- | --- | --- | --- | --- | --- | --- | --- | --- | --- | --- | --- | --- | --- | --- | --- | --- | --- | --- |
| 2015 | 21 | 27 | 20 | 26 | 24 | 26 | 24 | 18 | 24 | 27 | 25 | 12 | 24 | 26 | 25 | 23 | 26 | 25 | 21 | 23 | 28 | 20 | 25 | 29 | 26 | 25 | 0 | 25 | 22 | 27 | 25 |
| 2016 | 27 | 27 | 28 | 25 | 24 | 28 | 26 | 25 | 25 | 27 | 24 | 13 | 23 | 26 | 25 | 23 | 26 | 24 | 20 | 23 | 28 | 22 | 26 | 23 | 26 | 24 | 0 | 25 | 22 | 24 | 25 |
| 2017 | 29 | 27 | 27 | 25 | 26 | 28 | 24 | 22 | 26 | 25 | 23 | 26 | 23 | 26 | 23 | 26 | 27 | 12 | 23 | 22 | 24 | 24 | 28 | 26 | 26 | 26 | 0 | 25 | 21 | 23 | 25 |
| 2018 | 28 | 25 | 26 | 25 | 26 | 28 | 27 | 24 | 26 | 23 | 26 | 24 | 23 | 25 | 26 | 27 | 25 | 25 | 23 | 10 | 24 | 25 | 29 | 23 | 25 | 27 | 10 | 26 | 19 | 23 | 24 |
| 2019 | 28 | 28 | 26 | 22 | 28 | 27 | 26 | 21 | 20 | 29 | 6 | 26 | 26 | 24 | 27 | 28 | 26 | 25 | 2 | 6 | 29 | 24 | 24 | 27 | 29 | 28 | 0 | 23 | 22 | 22 | 27 |
| 2020 | 28 | 28 | 27 | 25 | 28 | 28 | 27 | 26 | 13 | 29 | 6 | 24 | 27 | 24 | 28 | 28 | 29 | 26 | 3 | 24 | 29 | 27 | 25 | 27 | 29 | 29 | 4 | 22 | 26 | 25 | 28 |
| 2021 | 28 | 28 | 27 | 24 | 29 | 29 | 28 | 27 | 29 | 29 | 27 | 28 | 29 | 27 | 28 | 29 | 28 | 29 | 13 | 25 | 29 | 27 | 29 | 28 | 29 | 29 | 18 | 23 | 27 | 27 | 28 |
| 2022 | 28 | 28 | 27 | 22 | 29 | 29 | 28 | 25 | 28 | 28 | 24 | 29 | 23 | 28 | 28 | 27 | 29 | 26 | 15 | 17 | 29 | 27 | 29 | 28 | 29 | 0 | 20 | 26 | 28 | 27 | 28 |

Note: Yellow color means the number of medicines is less than 15.

**Supplementary Table 2 National- level price of anticancer medicine from 2015 to 2022**

|  | Medicine | 2015 | 2016 | 2017 | 2018 | 2019 | 2020 | 2021 | 2022 |
| --- | --- | --- | --- | --- | --- | --- | --- | --- | --- |
| Non-targeted medicines | Busulfan | 6260.06 | 6137.36 | 6039.91 | 5910.55 | 5637.48 | 5488.23 | 5425.04 | 5307.59 |
|  | Ifosfamide | 99.27 | 101.25 | 99.32 | 100.86 | 48.29 | 46.19 | 91.53 | 56.53 |
|  | Cyclophosphamide | 3.71 | 6.87 | 6.84 | 13.61 | 13.36 | 30.60 | 29.86 | 29.74 |
|  | Doxorubicin | 15.85 | 14.89 | 14.43 | 14.55 | 12.06 | 11.69 | 12.35 | 16.19 |
|  | Daunorubicin | 14.10 | 14.07 | 13.00 | 12.20 | 12.34 | 9.97 | 9.10 | 8.71 |
|  | Etoposide | 7.01 | 6.88 | 5.67 | 5.46 | 2.38 | 2.55 | 7.53 | 7.84 |
|  | Homoharringtonine | 10.02 | 10.09 | 85.10 | 104.62 | 105.06 | 102.92 | 101.78 | 102.20 |
|  | Taxol | 153.13 | 144.38 | 145.34 | 138.26 | 78.04 | 105.11 | 67.86 | 85.68 |
|  | Vincristine | 3.53 | 5.11 | 11.72 | 16.59 | 17.86 | 31.20 | 71.17 | 66.15 |
|  | Letrozole | 22.79 | 21.11 | 21.06 | 19.69 | 17.24 | 14.32 | 7.67 | 4.82 |
|  | Tamoxifen | 0.82 | 0.93 | 1.01 | 1.06 | 1.16 | 1.28 | 2.48 | 1.83 |
|  | Gemcitabine | 253.10 | 241.94 | 222.40 | 189.13 | 154.54 | 111.77 | 94.08 | 53.81 |
|  | Fluorouracil | 9.37 | 10.07 | 19.66 | 28.80 | 28.16 | 23.38 | 22.61 | 21.48 |
|  | Cytarabine | 17.02 | 17.20 | 16.50 | 15.67 | 8.03 | 13.58 | 14.39 | 13.83 |
|  | Mercaptopurine | 2.27 | 2.08 | 2.06 | 6.85 | 6.09 | 6.21 | 6.10 | 5.78 |
|  | Hydroxyurea | 1.98 | 2.12 | 2.04 | 1.92 | 2.29 | 2.15 | 1.77 | 3.48 |
|  | Arsenous acid | 81.59 | 84.02 | 82.54 | 80.99 | 78.44 | 76.61 | 75.81 | 74.42 |
|  | Asparaginase | 78.66 | 75.80 | 70.69 | 69.31 | 69.59 | 62.89 | 63.85 | 61.52 |
|  | Oxaliplatin | 91.29 | 93.35 | 88.88 | 92.79 | 25.72 | 43.68 | 33.96 | 56.88 |
|  | Capecitabine | 209.15 | 188.05 | 153.57 | 137.38 | 83.16 | 83.72 | 29.95 | 24.60 |
|  | Carboplatin | 19.91 | 20.40 | 19.46 | 18.12 | 12.89 | 15.58 | 18.02 | 23.69 |
|  | Calcium folinate | 24.05 | 21.05 | 16.82 | 15.08 | 9.83 | 8.11 | 10.50 | 14.32 |
|  | Cisplatin | 7.82 | 7.66 | 8.28 | 6.37 | 4.96 | 4.98 | 5.27 | 5.23 |
| Targeted medicines | Rituximab | 1297.86 | 1272.75 | 1030.96 | 764.10 | 679.53 | 573.10 | 532.25 | 482.52 |
|  | Trastuzumab | 1215.00 | 1148.22 | 782.36 | 370.54 | 348.76 | 263.71 | 254.35 | 244.63 |
|  | Alimta | 579.67 | 559.06 | 493.08 | 427.56 | 292.88 | 277.68 | 261.19 | 129.15 |
|  | Imatinib | 538.51 | 382.63 | 276.19 | 228.68 | 127.15 | 106.11 | 78.68 | 48.93 |
|  | Icotinib | 148.98 | 131.90 | 101.07 | 72.72 | 68.30 | 65.93 | 64.97 | 39.91 |
|  | Gefitinib | 556.10 | 450.82 | 270.28 | 253.92 | 76.69 | 56.31 | 48.18 | 27.40 |

Note: The price is the cost per defined daily does (DDDC) for each medicine


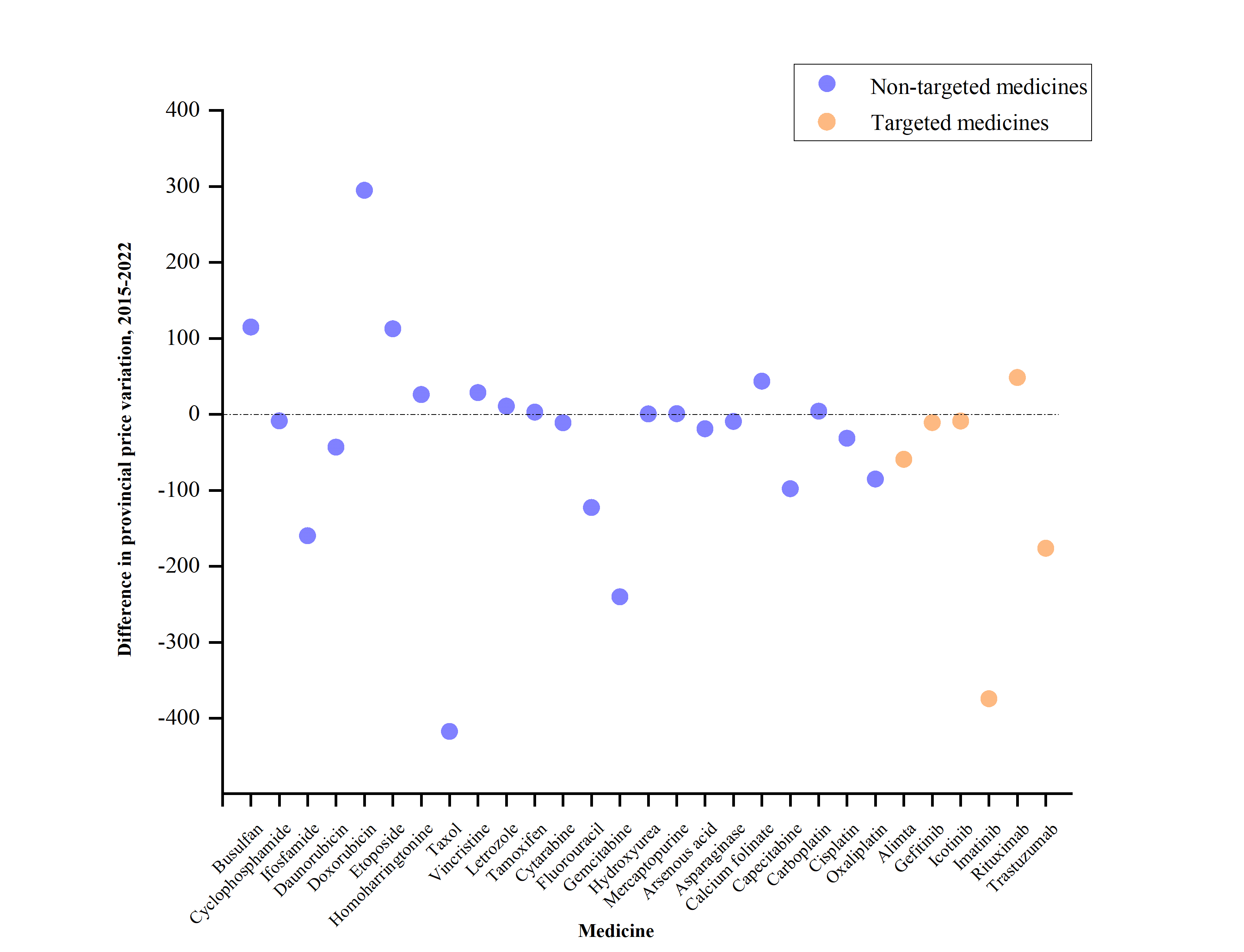


Supplementary Figure 1 Difference in provincial price variation of anticancer medicines between 2015 and 2022

Note: the difference equals to provincial price variation in 2022 minus that in 2015


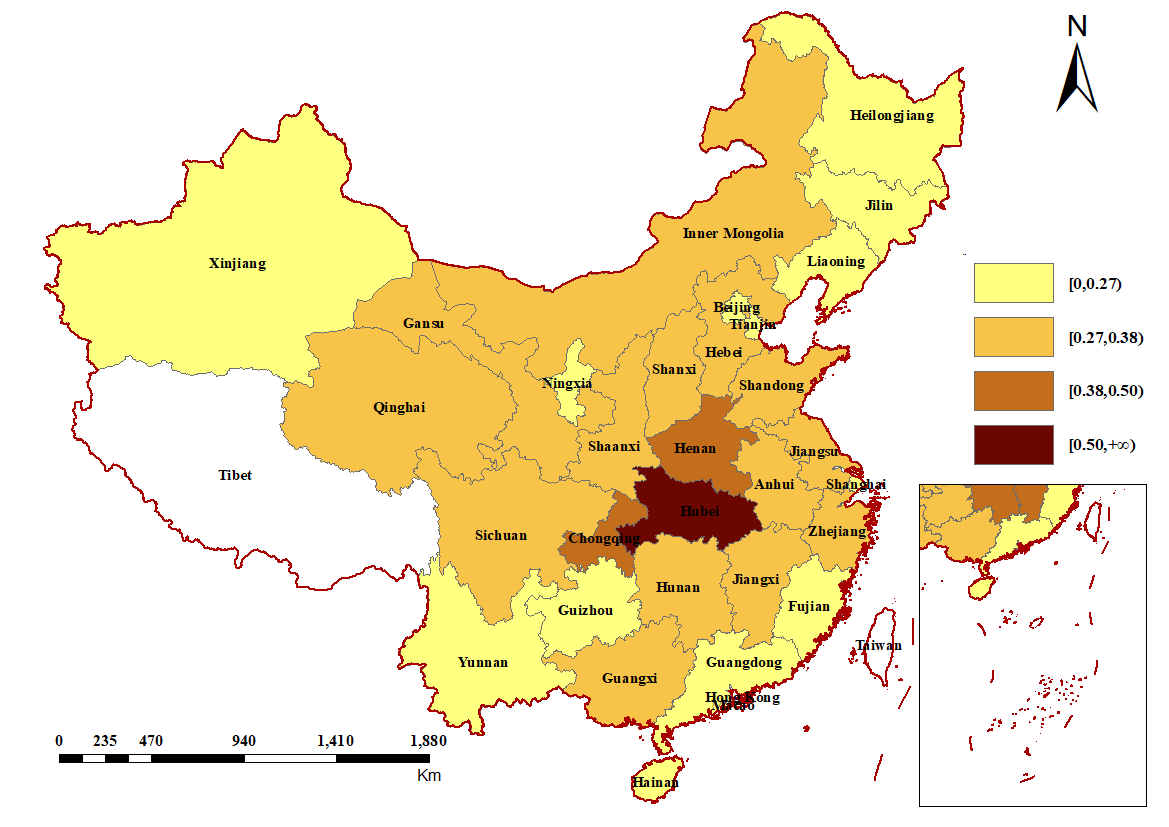


Supplementary Figure 2 Percentage of price-increased medicines among medicines whose procurement data were available in each province

Note: Tibet was excluded because of the low number of eligible drugs in that province.


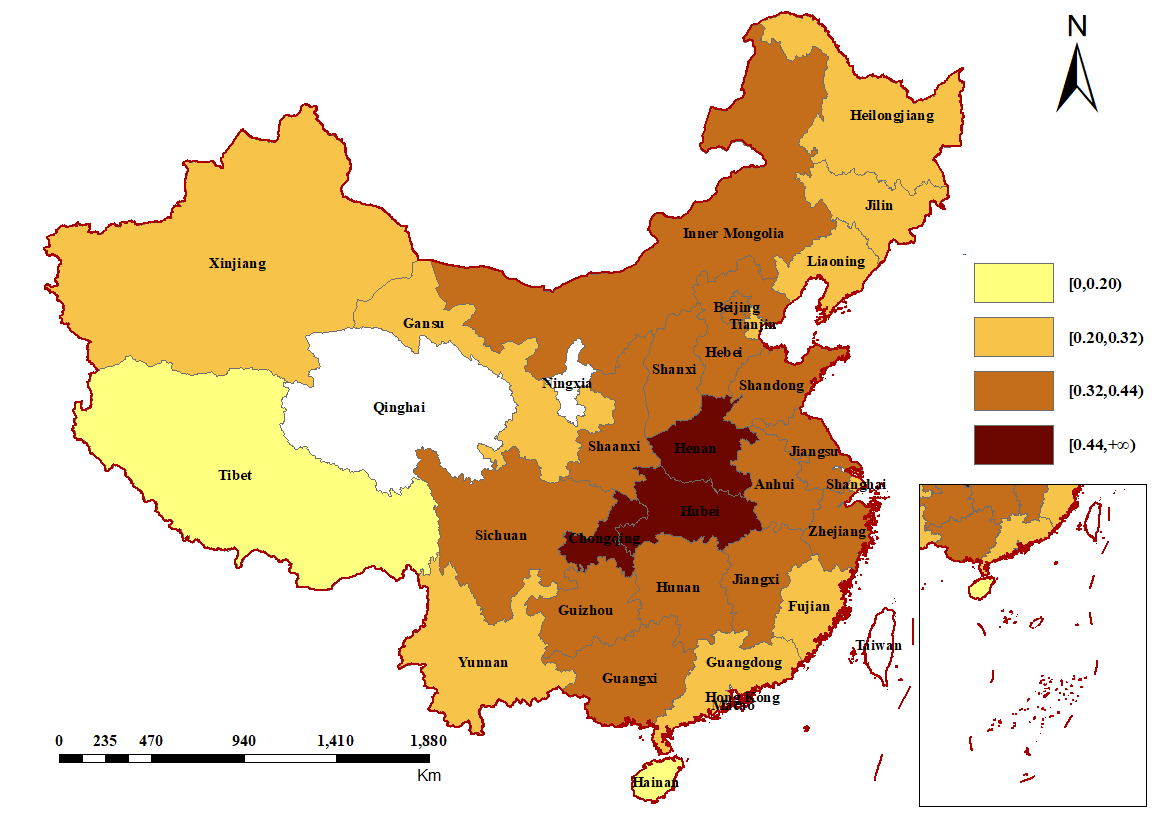


Supplementary Figure 3 Percentage of price-increased medicines among the medicine-province datasets for which procurement data were available in both 2015 and 2022

Note: Qinghai and Ningxia excluded because of the low number of eligible drugs in that province.
